# Supplementary material for: Multi-omics analysis identifies an M-MDSC-like immunosuppressive phenotype in lineage-switched AML with KMT2A rearrangement
Source: Nat Commun. 2025 Aug 26;16:7955. doi: 10.1038/s41467-025-63271-y (PMC12381044; doi:10.1038/s41467-025-63271-y)
Supplement: Supplementary file 1 — Supplementary Information [file 41467_2025_63271_MOESM1_ESM.pdf]

Multi-omics analysis identifies an M-MDSC-like immunosuppressive phenotype in lineage-switched AML with *KMT2A* rearrangement

Takashi Mikami, Itaru Kato, Junko Takita et. al.

Supplementary Information (SI)

1. **Supplementary figures**
   Supplementary Fig.1 Truncated *KMT2A* transcript observed in LS-AML5.
   Supplementary Fig.2 Supporting information for consensus clustering and the PCA plots.
   Supplementary Fig.3 Additional GSEA and copy number analysis.
   Supplementary Fig.4 Additional information about the analysis of leukemia cells by CyTOF.
   Supplementary Fig.5 Gating schemes used to detect of Tregs and their effector subsets cells by mass cytometry analysis.
   Supplementary Fig.6 Gating schemes used for sorting M-MDSC-like AML cells from LS AML samples.
   Supplementary Fig.7 Identification of the KMT2A::AFF1 genomic junction of M-MDSC-like AML cells (LS-AML3 and LS-AML4).
   Supplementary Fig.8 Pathway analysis using hematopoietic gene sets: M-MDSCs vs. non-MDSC myeloid cells in autoimmune diseases.
   Supplementary Fig.9 In vitro high-throughput drug sensitivity test.

2. **Supplementary tables**
   Supplementary Table 1 KMT2A::AFF1 fusion detected by RNA-seq in LS AML cases.
   Supplementary Table 2 Clinical characteristics of inhouse LS AML and LC AML cases.
   Supplementary Table 3 Sample names of deposited data (RNA-seq) used in the current study.
   Supplementary Table 4 List of M-MDSC-related genes used for differentially expressed gene analysis of LS AML compared with LC AML.
   Supplementary Table 5 CyTOF panel.
   Supplementary Table 6 Drugs used for the high throughput drug sensitivity test.
   Supplementary Table 7 Processing parameters used for SPRING analysis.
   Supplementary Table 8 Flow cytometry panel used for the Treg co-culture assay.

Supplementary Figure 1

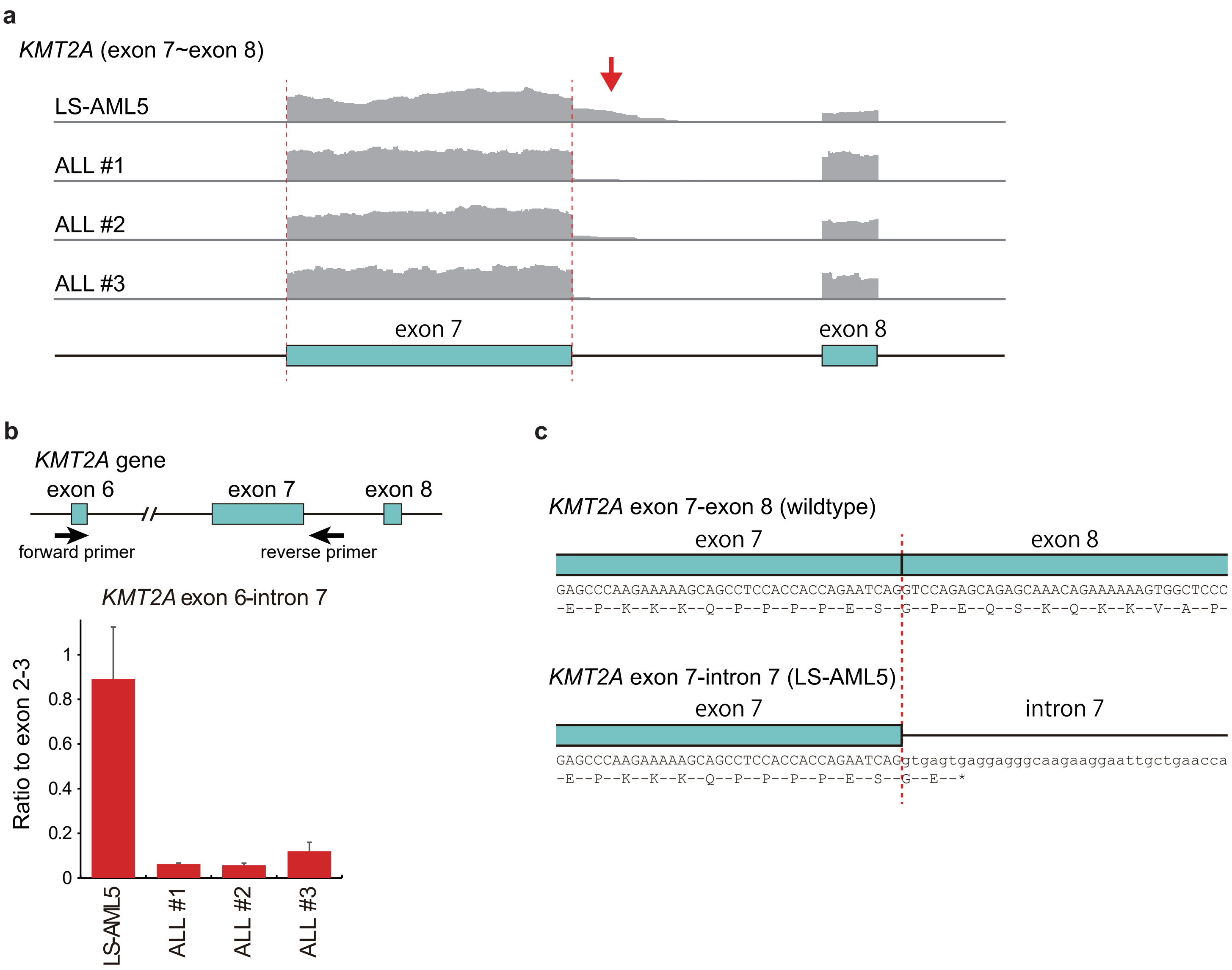

Truncated *KMT2A* transcript observed in LS-AML5.

a Read depth of exon 7 and exon 8 of *KMT2A* transcript in LS-AML5 obtained by RNA-seq visualized in coverage track of Integrative genomic viewer (IGV) are shown. The results from three ALL samples with germline *KMT2A* are shown for comparison. Reads containing intron 7 of *KMT2A* were frequently observed in LS-AML5 (red arrow). **b** Aberrant transcript of *KMT2A* containing intron 7 was measured by reverse transcription-quantitative polymerase chain reaction (RT-qPCR). Forward primer was designed within exon 6 and reverse primer was designed downstream of splice donor sequence of intron 7 of *KMT2A* (upper panel). Results of RT-qPCR are shown in lower panel. The aberrant transcript containing exon7 which was directly connected to intron 7 sequence was highly expressed in LS-AML5 compared to other ALL samples. Source data are provided as a Source Data file. **c** Nucleotide sequence and expected coding amino acids of KMT2A exon 7-intron 7 aberrant transcripts are shown. Aberrant protein translation terminates two amino acids after the end of exon 7 of *KMT2A* due to termination codon provided by intron 7. This results in truncated N-terminal only KMT2A protein in LS-AML5.

Supplementary Figure 2

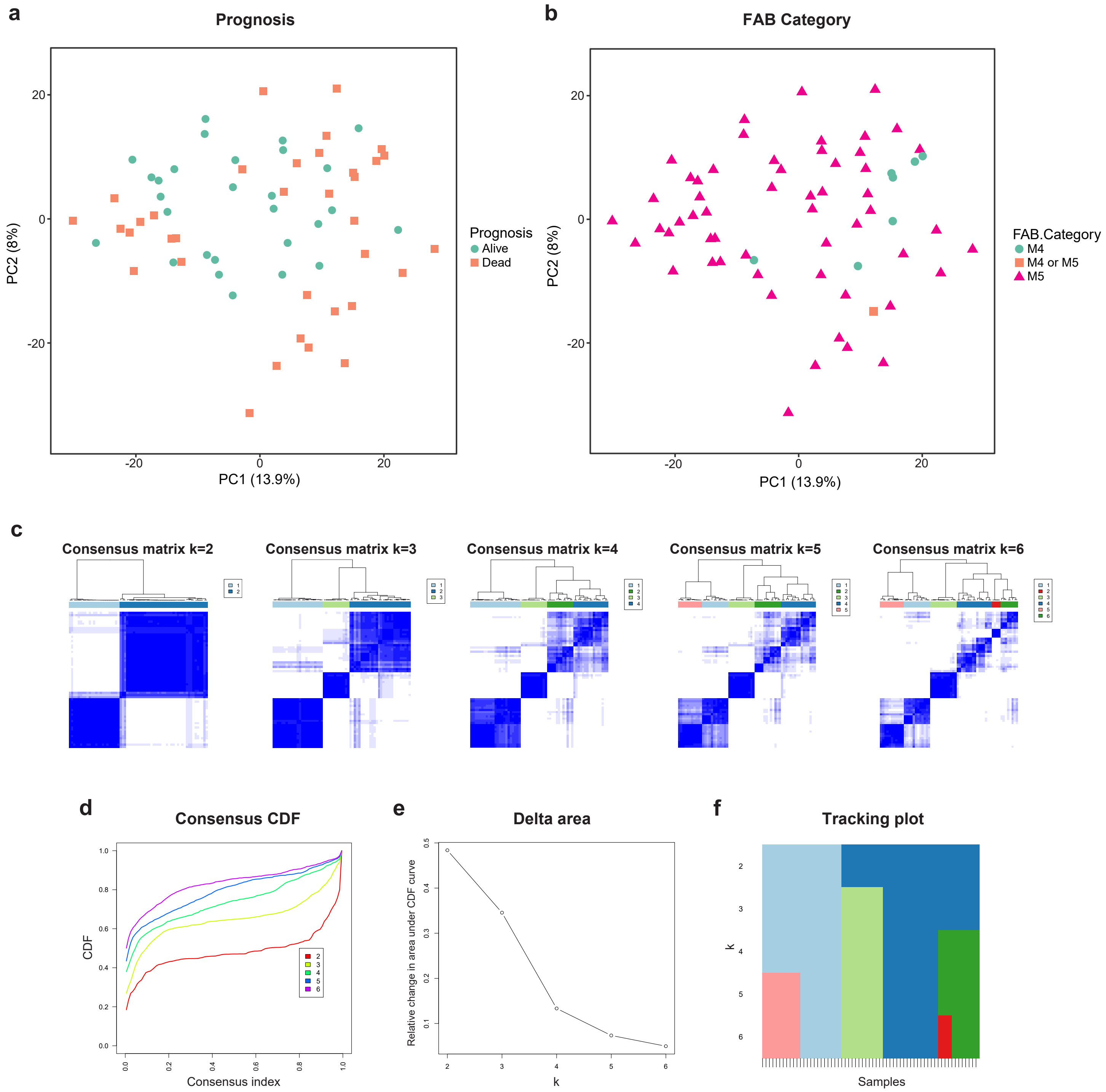

Supporting information for consensus clustering and the PCA plots.

a, b PCA distribution plots indicating prognosis (a) and FAB category (b) in LS AML and LC AML samples. Source data are provided as a Source Data file. **c, d, e, f** Consensus matrix plots for each k (c), an empirical cumulative distribution function (CDF) plot displaying consensus distribution (d), a delta area plot (e), and an item tracking plot (f). All were calculated by ConsensusClusterPlus to determine the optimal number of clusters. In this case, k=3 (3 clusters) gave the most stable result.

Supplementary Figure 3

a

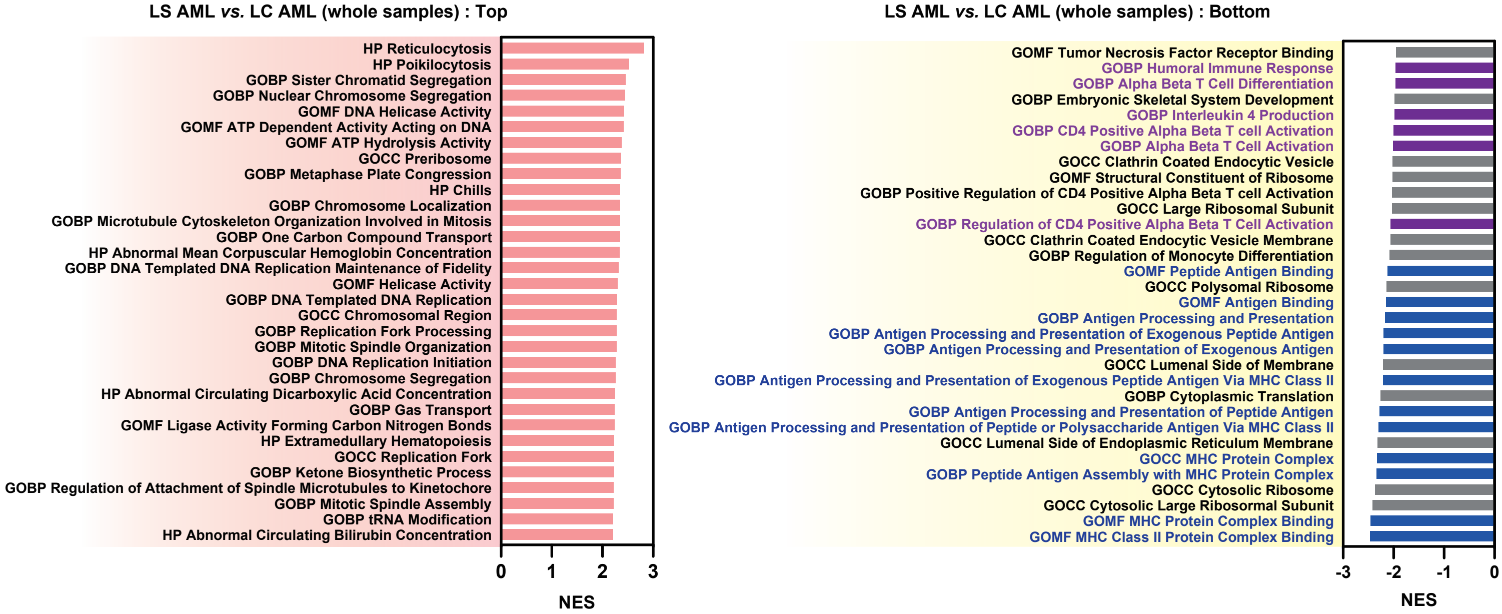

b

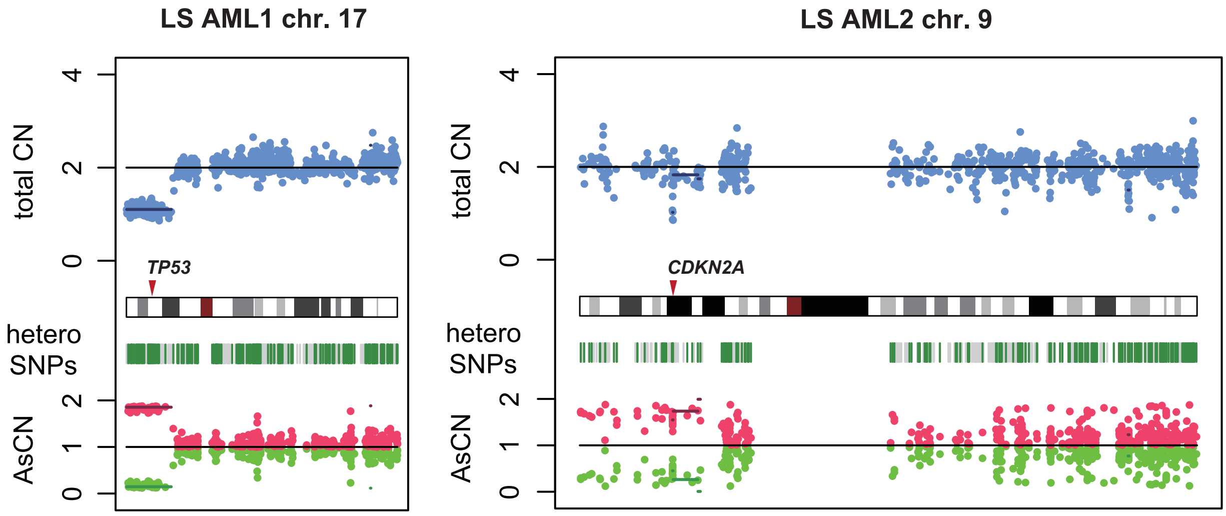

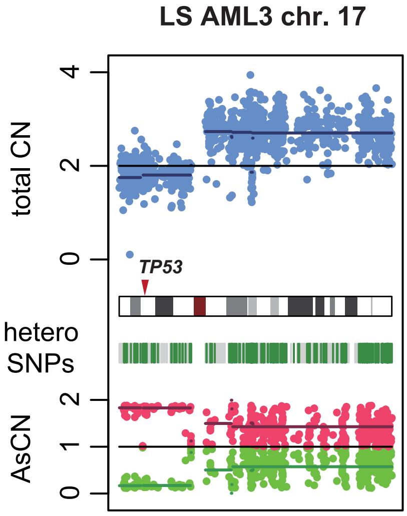

Additional GSEA and copy number analysis.

a Ontology gene sets ranked in the top and bottom normalized enrichment scores (NESs) calculated by gene set enrichment analysis (GSEA). LS AML and LC AML (whole samples) were compared. Adaptive immune-related gene sets are shown in purple; antigen presentation-related gene sets are shown in blue. GOCC: gene ontology cellular component; GOBP: gene ontology biological process; GOMF: gene ontology molecular function; HP: human phenotype. cells. Source data are provided as a Source Data file. **b** Copy number (CN) analysis for LS AML1, 2 and 3. chr: chromosome; SNP: single nucleotide polymorphism; As: allele-specific.

Supplementary Figure 4

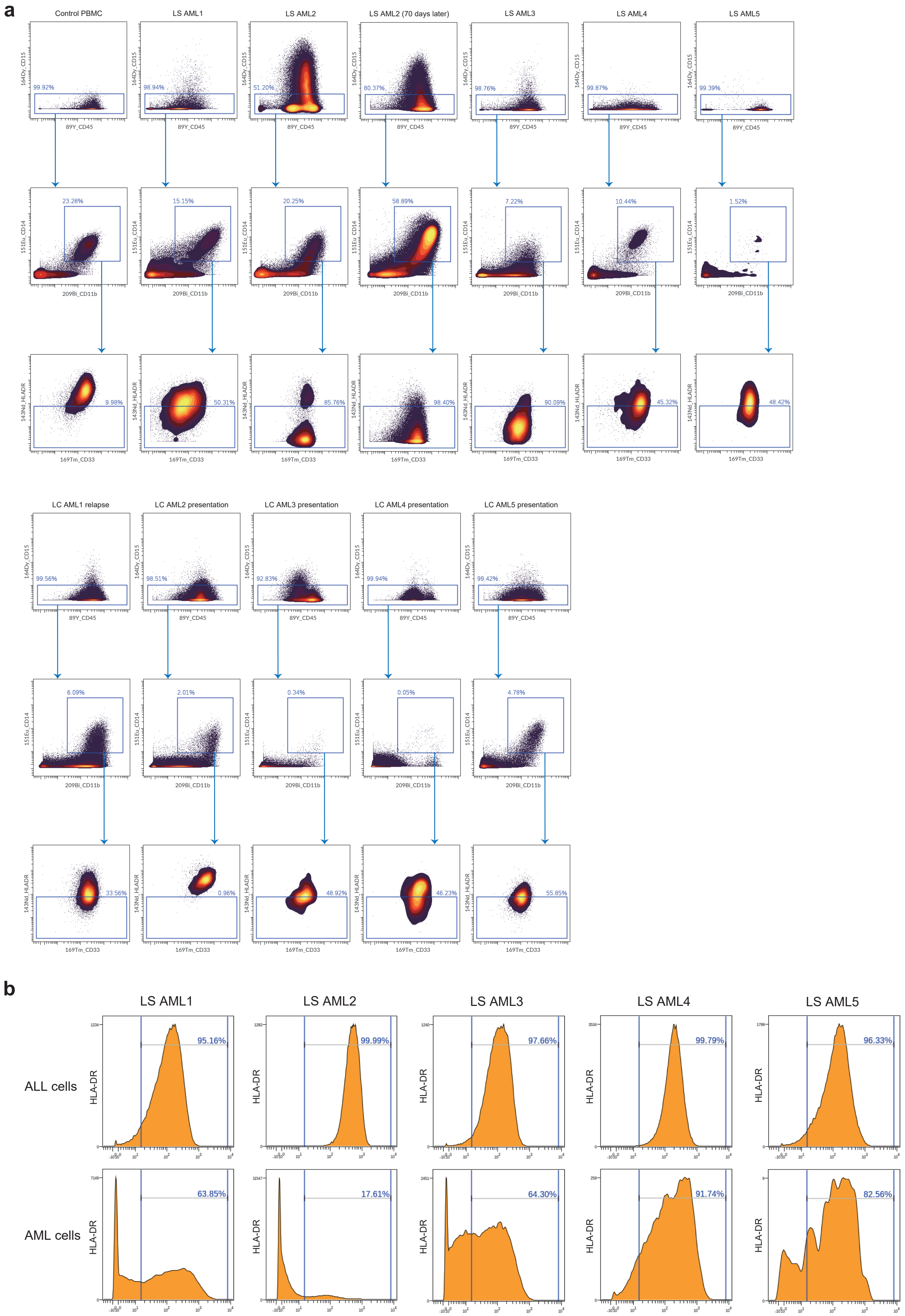

Additional information about the analysis of leukemia cells by CyTOF.

a Serial gating schemes used to detect of M-MDSC-like AML cells by mass cytometry analysis.

b HLA-DR expression on ALL cells and AML cells in each LS patient.

Supplementary Figure 5

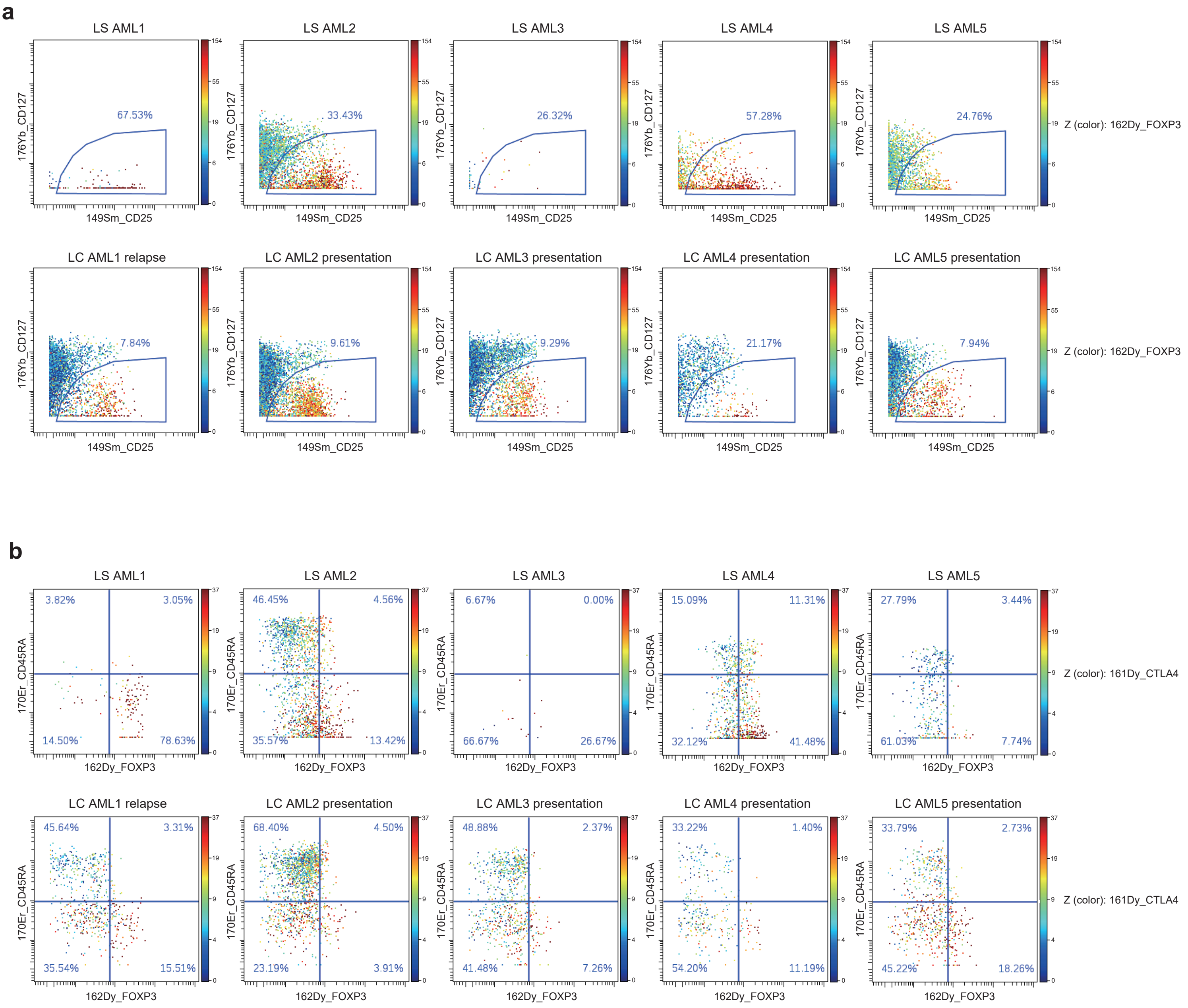

Gating schemes used to detect of Tregs and their effector subsets cells by mass cytometry analysis.

a Gating schemes for detecting Tregs ($CD4^+CD25^+CD127^-$ T cells) in $CD4^+$ T cells. **b** Gating schemes for detecting effector Tregs ($CD4^+CD25^+CD127^-CD45RA^-Foxp3^{high}$ T cells) in Tregs.

Supplementary Figure 6

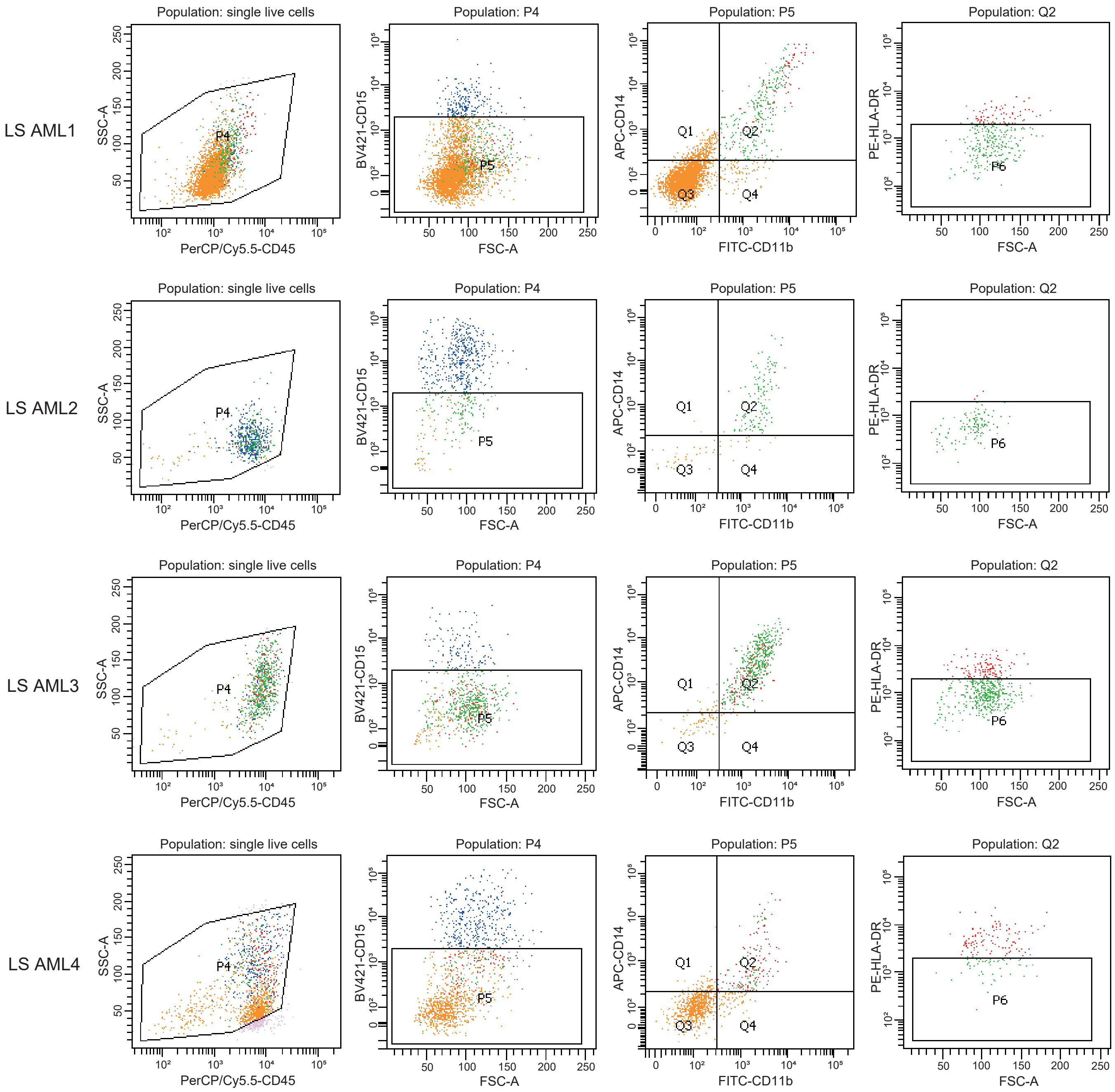

Gating schemes used for sorting M-MDSC-like AML cells from LS AML samples.

Supplementary Figure 7

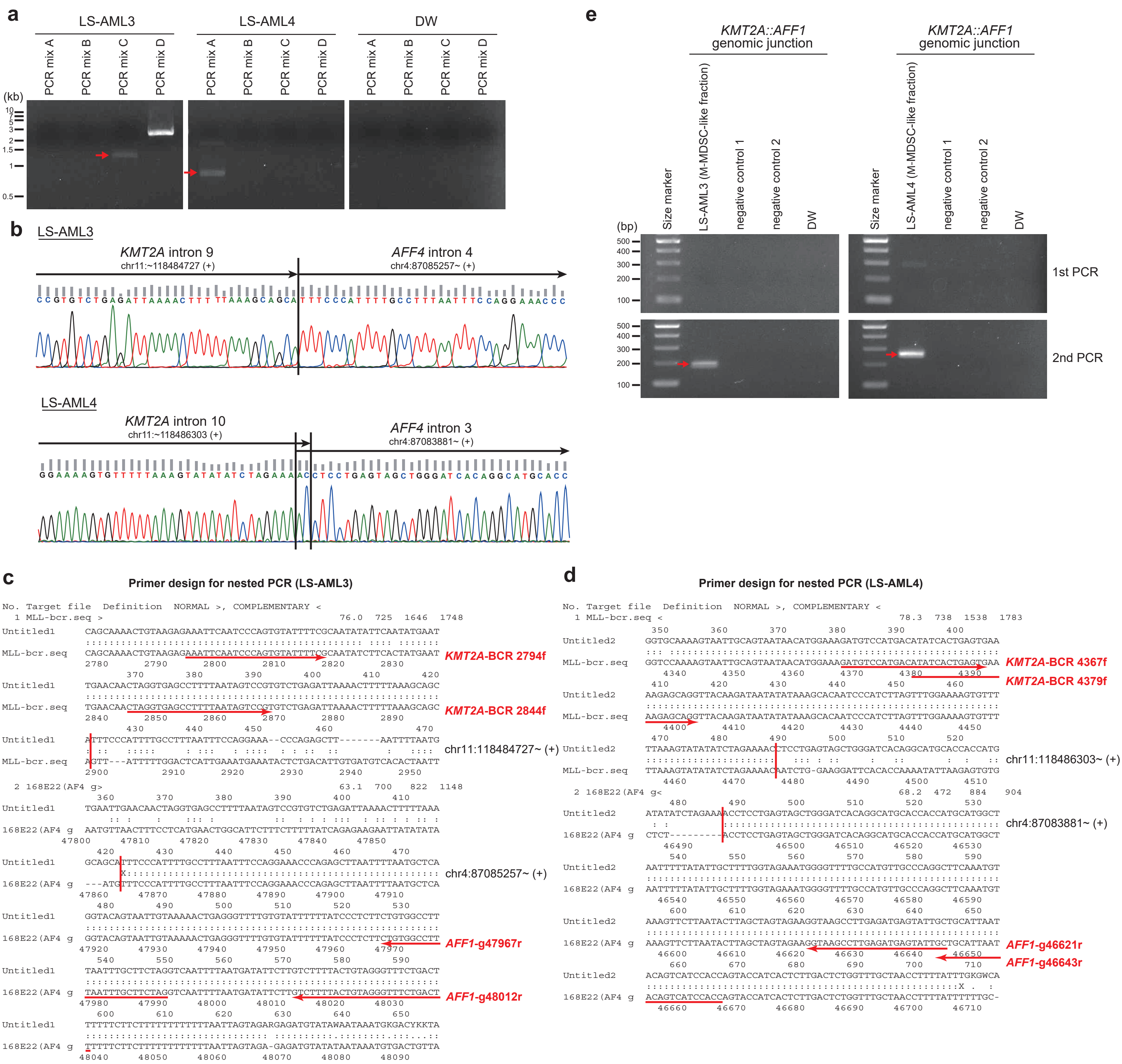

Identification of the *KMT2A::AFF1* genomic junction of M-MDSC-like AML cells (LS-AML3 and LS-AML4).

a PCR amplification of the *KMT2A::AFF1* genomic sequences performed with four separated tubes (PCR mix A to D) each containing one of *AFF1* reverse primer sets and 19 *KMT2A* forward primers. Nineteen forward primers were designed every 300 to 700 basepairs to cover the whole breakpoint cluster region of *KMT2A* and mixture of all primers were used for PCR. As for *AFF1*, 48 reverse primers were designed every 700 bps to 1.5 kb to cover exon 3 to exon 7 of *AFF1* and separated into four groups of primer sets (A to D) for PCR amplification. Red arrows indicate successfully amplificated *KMT2A::AFF1* genomic sequences from primary (disease onset, acute lymphoblastic leukemia) samples of LS-AML3 and LS-AML4. DW: distilled water. An unedited gel image is provided as a Source Data file. **b** *KMT2A::AFF1* genomic sequences identified by Sanger sequencing using amplificated PCR products from primary samples of LS-AML3 and LS-AML4. **c, d** Primer pairs for the nested PCR amplification were designed in reference to the *KMT2A::AFF1* genomic sequences obtained from primary samples of LS-AML3 (c) and LS-AML4 (d). Primer pairs for each case are indicated as red arrows, and each break point region is shown as a red vertical line. **e** Nested PCR amplification of the *KMT2A::AFF1* genomic sequences of M-MDSC-like fraction sorted from LS AML samples (LS-AML3 and LS-AML4). Red arrows indicate successfully amplificated *KMT2A::AFF1* genomic sequences, which were confirmed to be identical to that of the primary samples. Unedited gel images are provided as a Source Data file.

Supplementary Figure 8

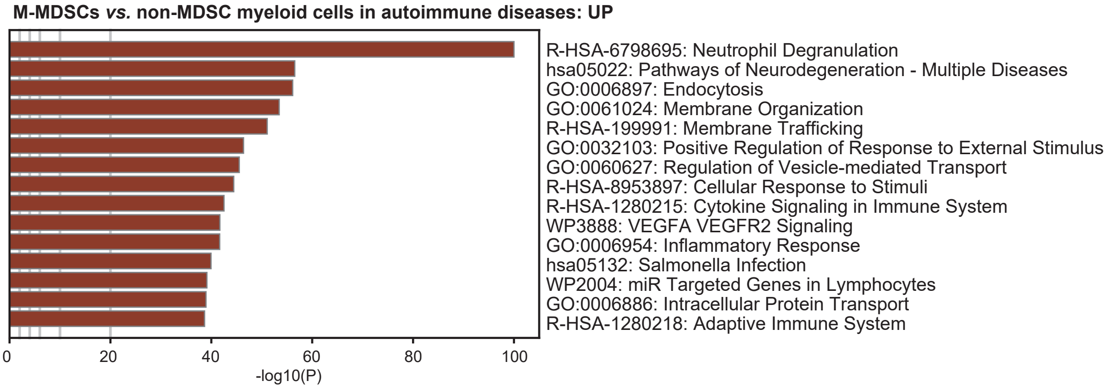

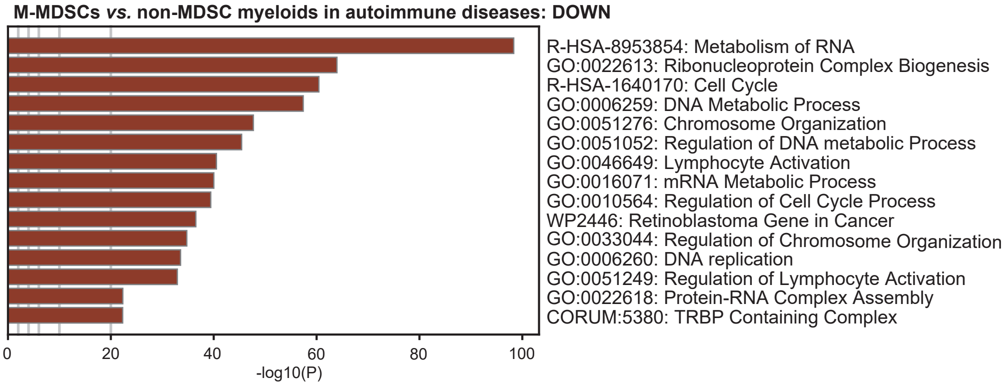

Pathway analysis using hematopoietic gene sets: M-MDSCs *vs.* non-MDSC myeloid cells in autoimmune diseases. M-MDSCs and non-MDSC myeloids in autoimmune diseases (n=3) were compared using hematopoietic gene set analysis. Enriched terms across input gene lists are ranked according to their adjusted p-values. R-HSA: reactome-Homo sapiens; hsa: homo sapiens (Kyoto Encyclopedia of Genes and Genomes: KEGG); GO: gene ontology; CORUM: comprehensive resource of mammalian protein complexes; WP: WikiPathways. Source data are provided as a Source Data file.

Supplementary Figure 9

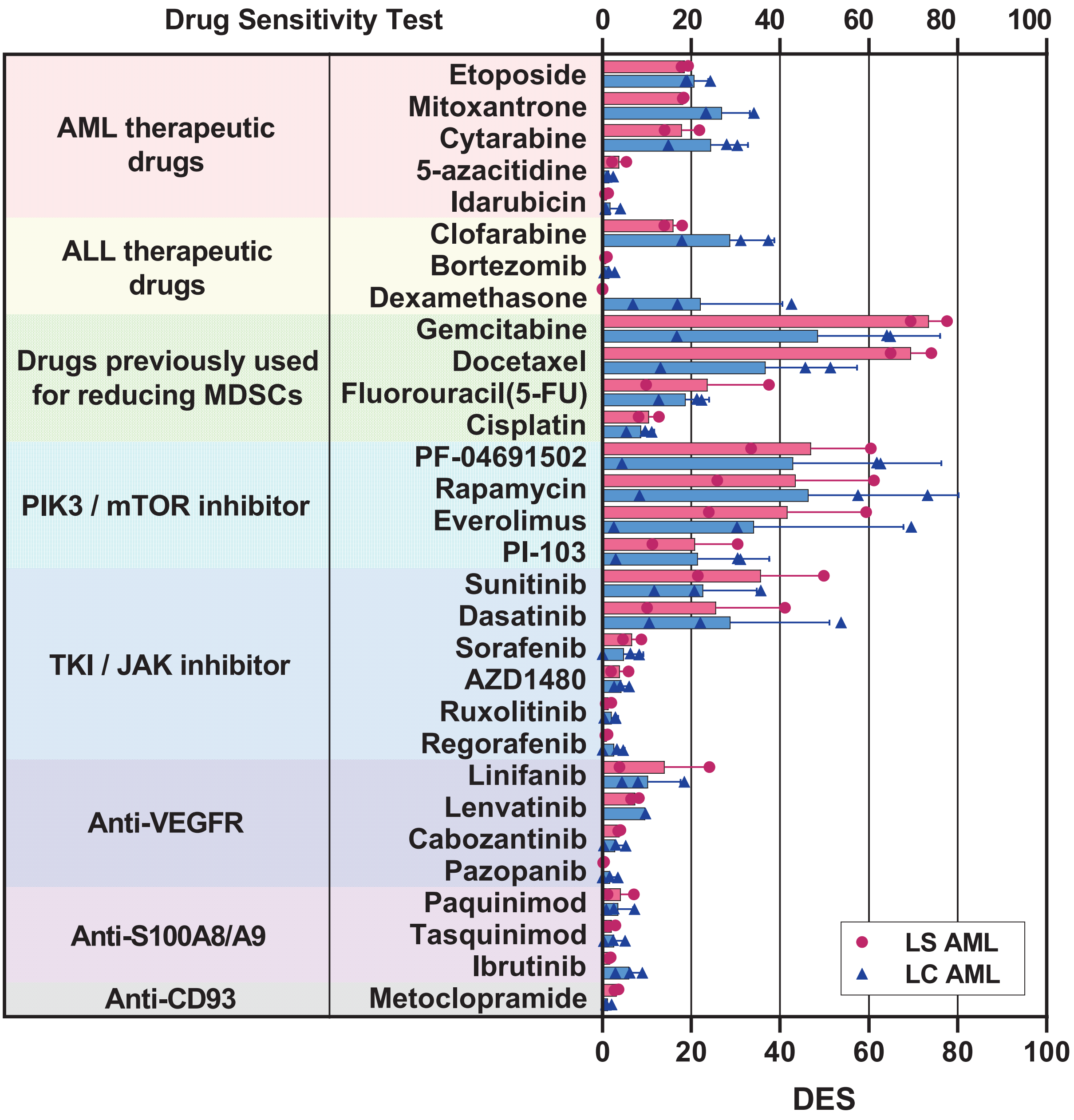

In vitro high-throughput drug sensitivity test.

Drug sensitivity scores (DESs) for LS AML and LC AML exposed to 30 anti-leukemic drugs. The scores were calculated from the cell viability measured after a 4-day incubation with serially diluted drugs. The bar represents the mean, and the error bar indicates standard deviation. The samples used in this experiment were as follows: LS AML1, LS AML2, LC AML1(relapse), LC AML3, and LC AML4. Source data are provided as a Source Data file.

Supplementary Table 1. *KMT2A::AFF1* fusion detected by RNA-seq in LS AML cases

Sample	5' chromosome	5' position	5' gene	3' chromosome	3' position	3' gene	Paired reads
LS AML1 presentation ALL	Chromosome 11	118355029	*KMT2A*	Chromosome 4	88005272	*AFF1*	18
LS AML1 relapse AML	Chromosome 11	118355029	*KMT2A*	Chromosome 4	88005272	*AFF1*	5
LS AML1 relapse AML (M-MDSC-like fraction)	Chromosome 11	118355029	*KMT2A*	Chromosome 4	88005272	*AFF1*	6
LS AML2 presentation ALL	Chromosome 11	118359329	*KMT2A*	Chromosome 4	87968746	*AFF1*	59
LS AML2 relapse AML	Chromosome 11	118359329	*KMT2A*	Chromosome 4	87968746	*AFF1*	7
LS AML2 relapse AML (M-MDSC-like fraction)	Chromosome 11	118359329	*KMT2A*	Chromosome 4	87968746	*AFF1*	7
LS AML3 presentation ALL	Chromosome 11	118355029	*KMT2A*	Chromosome 4	88011136	*AFF1*	43
LS AML3 relapse AML	Chromosome 11	118355029	*KMT2A*	Chromosome 4	88011136	*AFF1*	11
LS AML4 presentation ALL	Chromosome 11	118359329	*KMT2A*	Chromosome 4	87968746	*AFF1*	10
LS AML4 relapse AML	Chromosome 11	118359329	*KMT2A*	Chromosome 4	87968746	*AFF1*	19

Supplementary Table 2. Clinical characteristics of inhouse LS AML and LC AML cases

Case	Sex	Age (year)	WCC($\times 10^9$/L)	Rearrangement	CNS	Treatment Protocol	Relapse	Time from initial diagnosis to lineage switching (day)	HSCT or Immunotherapy prior to lineage switching	FAB Category	Prognosis
LS AML1	Male	10 - 12	997.0	*KMT2A::AFF1*	Negative	JPLSG ALL-B12	Yes	205	Blinatumomab	M5 (after lineage switching)	Dead
LS AML2	Female	0 - 0.5	4.9	*KMT2A::AFF1*	Negative	JPLSG MLL-17	Yes	79	None	M5 (after lineage switching)	Dead
LS AML3	Male	0.5 - 1	141.2	*KMT2A::AFF1*	Negative	JPLSG MLL-17	Yes	417	Blinatumomab, CD19 CAR-T(Tisagenlecleucel)	M5 (after lineage switching)	Dead
LS AML4	Female	0 - 0.5	200	*KMT2A::AFF1*	Negative	JPLSG MLL-17	Yes	402	HSCT	M5 (after lineage switching)	Dead
LS AML5	Male	10 - 12	10.9	Truncated transcript of *KMT2A* containing intron 7	Negative	JPLSG ALL-B19	Yes	771	None	M5 (after lineage switching)	Dead
LC AML1	Male	0 - 0.5	15.1	*KMT2A::MLLT10*	Negative	JPLSG AML-05	Yes	NA	NA	M5	Alive
LC AML2	Female	1 - 3	7.2	*KMT2A::MLLT3*	Negative	JPLSG AML-12	No	NA	NA	M5	Alive
LC AML3	Male	1 - 3	70.5	*KMT2A::MLLT11*	Positive	JACLS AML99	No	NA	NA	M4	Alive
LC AML4	Female	13 - 15	102.0	*KMT2A::MLLT1*	Negative	JPLSG AML-12	No	NA	NA	M5	Alive
LC AML5	Female	1 - 3	13.3	*KMT2A::SEPT9*	Negative	JACLS AML99	No	NA	NA	M4	Alive

WCC: white cell count; CNS: central nevous system; HSCT: hematopoietic stem cell transplantation; FAB: French-American-British classification; CAR-T: chimeric antigen receptor-T cell therapy

JPLSG: Japanese Pediatric Leukemia/Lymphoma Study Group; JACLS: Japan Association of Childhood Leukemia Study

Supplementary Table 3. Sample names of deposited data (RNA-seq) used in the current study

Data resource	Status	Sample name
TARGET	presentation	TARGET_20_PAEAKL_09A_03R
TARGET	presentation	TARGET_20_PAEGRI_03A_01R
TARGET	presentation	TARGET_20_PAEIKD_09A_01R
TARGET	presentation	TARGET_20_PAKIYW_09A_01R
TARGET	presentation	TARGET_20_PAKLPD_09A_02R
TARGET	presentation	TARGET_20_PAKTCX_09A_02R
TARGET	presentation	TARGET_20_PANFMG_09A_04R
TARGET	presentation	TARGET_20_PANPKN_09A_03R
TARGET	presentation	TARGET_20_PANZKA_09A_02R
TARGET	presentation	TARGET_20_PAPWZR_09A_01R
TARGET	presentation	TARGET_20_PAPZIZ_09A_03R
TARGET	presentation	TARGET_20_PARASV_03A_03R
TARGET	presentation	TARGET_20_PARBIU_09A_03R
TARGET	presentation	TARGET_20_PARBRA_09A_03R
TARGET	presentation	TARGET_20_PARBXE_09A_04R
TARGET	presentation	TARGET_20_PARFGK_09A_01R
TARGET	presentation	TARGET_20_PARJCR_09A_01R
TARGET	presentation	TARGET_20_PARKCX_09A_03R
TARGET	presentation	TARGET_20_PARKFB_09A_02R
TARGET	presentation	TARGET_20_PARWDZ_09A_01R
TARGET	presentation	TARGET_20_PARXZP_09A_01R
TARGET	presentation	TARGET_20_PASCGR_03A_02R
TARGET	presentation	TARGET_20_PASDGX_09A_02R
TARGET	presentation	TARGET_20_PASEFD_09A_01R
TARGET	presentation	TARGET_20_PASFNP_09A_02R
TARGET	presentation	TARGET_20_PASGGK_03A_01R
TARGET	presentation	TARGET_20_PASLSD_03A_01R
TARGET	presentation	TARGET_20_PASMGW_09A_01R
TARGET	presentation	TARGET_20_PASNIY_09A_02R
TARGET	presentation	TARGET_20_PASPFE_09A_01R
TARGET	presentation	TARGET_20_PASSSI_09A_02R
TARGET	presentation	TARGET_20_PASSWG_03A_01R
TARGET	presentation	TARGET_20_PASTUH_03A_01R
TARGET	presentation	TARGET_20_PASVVS_09A_03R
TARGET	presentation	TARGET_20_PASVYL_09A_01R
TARGET	presentation	TARGET_20_PASZLJ_09A_01R
TARGET	presentation	TARGET_20_PATDNN_09A_03R
TARGET	presentation	TARGET_20_PATJHJ_09A_01R
TARGET	presentation	TARGET_21_PASIGA_09A_01R
TARGET	presentation	TARGET_21_PATAIJ_09A_01R
TARGET	relapse	TARGET_20_PAEIKD_04A_01R
TARGET	relapse	TARGET_20_PAKTCX_04A_01R
TARGET	relapse	TARGET_20_PARBIU_04A_02R
TARGET	relapse	TARGET_20_PASCGR_04A_01R
TARGET	relapse	TARGET_20_PASGZS_04A_01R
TARGET	relapse	TARGET_20_PASTUH_04A_01R
TARGET	relapse	TARGET_20_PASVVS_04A_01R
TARGET	relapse	TARGET_20_PATDNN_04A_02R
TARGET	relapse	TARGET_20_PATJHJ_40A_01R
Blood 2022	lineage switch	LS01_relapse_AML
Blood 2022	lineage switch	LS04_relapse_AML
Blood 2022	lineage switch	LS05_relapse_AML
Blood 2022	lineage switch	LS06_relapse_AML

Supplementary Table 4. List of M-MDSC-related genes used for differentially expressed gene analysis of LS AML compared with LC AML

S100A9
S100A8
ARG1
ARG2
NOS2
IL10
VEGFA
WFDC17
CD14
TGFB1
TNF
STAT3
IL6
ORM1
S100P
SLCO4C1
CYP1B1
CLEC4D
CHD7
CKAP4
MSRB1
CLU
CD82
CPEB4
TLK1
OSBPL8
ABHD2
ATP6V1E1
ROCK1
CD84
CXCR1
CXCL1
PTGES

Supplementary Table 5. CyTOF panel

Metal Label	Target	Clone	Vendor	Note
89Y	CD45	HI30	Standard BioTools	
115In	CD235ab	HIR2	BioLegend	Self conjugation
139La	CD86	BU63	BioLegend	Self conjugation
141Pr	CD196 (CCR6)	G034E3	Standard BioTools	
142Nd	CD19	HIB19	Standard BioTools	
143Nd	HLA-DR	L243	Standard BioTools	
144Nd	CD38	HIT2	Standard BioTools	
145Nd	CD4	RPA-T4	Standard BioTools	
146Nd	CD8a	RPA-T8	Standard BioTools	
147Sm	CD20	2H7	Standard BioTools	
148Nd	CD274 (PD-L1)	29E.2A3	Standard BioTools	
149Sm	CD25 (IL-2R)	2A3	Standard BioTools	
150Nd	CD134 (OX40)	ACT35	Standard BioTools	
151Eu	CD14	M5E2	Standard BioTools	
152Sm	CD13	WM15	Standard BioTools	
153Eu	TIM-3	F38-2E2	Standard BioTools	
154Sm	CD3	UCHT1	Standard BioTools	
155Gd	CD27	L128	Standard BioTools	
156Gd	CD183 (CXCR3)	G025H7	Standard BioTools	
158Gd	CD10	HI10a	Standard BioTools	
159Tb	CD22	HIB22	Standard BioTools	
160Gd	CD28	CD28.2	Standard BioTools	
161Dy	CD152 (CTLA-4)	14D3	Standard BioTools	Intracellular staining
162Dy	Foxp3	236A/E7	eBioscience	Self conjugation, Intracellular staining
163Dy	CD56 (NCAM)	NCAM16.2	Standard BioTools	
164Dy	CD15 (SSEA-1)	W6D3	Standard BioTools	
165Ho	CD223 (LAG-3)	11C3C65	Standard BioTools	
166Er	CD34	581	Standard BioTools	
167Er	CD197 (CCR7)	G043H7	Standard BioTools	
168Er	CD357 (GITR)	621	BioLegend	Self conjugation
169Tm	CD33	WM53	Standard BioTools	
170Er	CD45RA	HI100	Standard BioTools	
171Yb	CD185 (CXCR5)	RF8B2	Standard BioTools	Self conjugation
172Yb	CD273 (PD-L2)	24F.10C12	Standard BioTools	
173Yb	CD137 (4-1BB)	4B4-1	Standard BioTools	
174Yb	CD279 (PD-1)	EH12.2H7	Standard BioTools	
175Lu	CD194 (CCR4)	L291H4	Standard BioTools	
176Yb	CD127 (IL-7Ra)	A019D5	Standard BioTools	
209Bi	CD11b (Mac-1)	ICRF44	Standard BioTools	

Supplementary Table 6. Drugs used for the high throughput drug sensitivity test

Drug	Final Concentration	Vendor
Gemcitabine Hydrochloride	10 μg/ml	Selleck Chemicals
Docetaxel	1 μg/ml	Selleck Chemicals
Fluorouracil (5-FU)	100 μM	Selleck Chemicals
Cisplatin	1 μg/ml	FUJIFILM Wako Pure Chemical Corporation
PF-04691502	1 μM	Selleck Chemicals
Rapamycin	1 μM	Selleck Chemicals
Everolimus	1 μM	Selleck Chemicals
PI-103	1 μM	Selleck Chemicals
Sunitinib	5 μM	Selleck Chemicals
Dasatinib	1 μM	Selleck Chemicals
Sorafenib	1 μM	Selleck Chemicals
AZD1480	1 μM	Selleck Chemicals
Ruxolitinib	1 μM	Selleck Chemicals
Linifanib	1 μM	Selleck Chemicals
Lenvatinib	1 μM	Selleck Chemicals
Cabozantinib	1 μM	Selleck Chemicals
Pazopanib	1 μM	Selleck Chemicals
Paquinimod	150 μM	Selleck Chemicals
Tasquinimod	50 μM	Selleck Chemicals
Ibrutinib	1 μM	Selleck Chemicals
Metoclopramide	5 μM	Selleck Chemicals
Etoposide	20 μg/ml	Selleck Chemicals
Mitoxantrone	500 ng/ml	Selleck Chemicals
Cytarabine	1 μg/ml	Selleck Chemicals
Clofarabine	100 ng/ml	Selleck Chemicals
Dexamthesone	100 μg/ml	Sigma Aldrich
5-azacitidine	1 μg/ml	Selleck Chemicals
Regorafenib	1 μM	Selleck Chemicals
Bortezomib	50 ng/ml	Selleck Chemicals
Idarubicin Hydrochloride	10 ng/ml	Selleck Chemicals

Supplementary Table 7. Processing parameters used for SPRING analysis

Number of PCA dimensions = 50

Gene filtering - minimum counts = 3

Gene filtering - gene variability percentile = 80.0

Number of nearest neighbors = 5

Cell filtering - minimum counts = 1000.0

Gene filtering - minimum cells = 3.0

Subplot method

Min expressing cells (gene filtering): 3

Min number of UMIs (gene filtering): 3.0

Gene variability percentile (gene filtering): 90.0

Number of principal components: 20

Number of nearest neighbors: 3

Number of force layout iterations: 500

Supplementary Table 8. Flow cytometry panel used for the Treg co-culture assay

Fluorochrome	Target	Clone	Note	Vendor	Concentration
FITC	CD4	RPA-T4		BioLegend	1:100
BV421	CD25	2A3		BD	2:100
PerCP/Cy5.5	CD127	A019D5		BioLegend	1:100
APC	CD45RA	HI100		BioLegend	1:100
eFluor 780			Fixable Viability Dye	eBioscience	1:2000
PE	FOXP3	236A/E7	Intracellular staining	Invitrogen	5:100
