# Supplementary material for: Multi-omics analysis identifies an M-MDSC-like immunosuppressive phenotype in lineage-switched AML with KMT2A rearrangement
Source: Nat Commun. 2025 Aug 26;16:7955. doi: 10.1038/s41467-025-63271-y (PMC12381044; doi:10.1038/s41467-025-63271-y)
Supplement: Supplementary file 2 — Description of Additional Supplementary Files [file 41467_2025_63271_MOESM2_ESM.pdf]

Multi-omics analysis identifies an M-MDSC-like immunosuppressive phenotype in lineage-switched AML with *KMT2A* rearrangement

Takashi Mikami, Itaru Kato, Junko Takita et. al.

Description of supplementary files

Supplementary Data 1

A GSEA report for LS AML vs LC AML in Cluster 2: Enrichment in LS AML. Ontology gene sets are listed in descending order of Normalized enrichment score (NES).

Supplementary Data 2

A GSEA report for LS AML vs LC AML in Cluster 2: Enrichment in LC AML. Ontology gene sets are listed in descending order of NES.

Supplementary Data 3

A GSEA report for LS AML vs LC AML (whole samples): Enrichment in LS AML. Ontology gene sets are listed in descending order of NES.

Supplementary Data 4

A GSEA report for LS AML vs LC AML (whole samples): Enrichment in LC AML. Ontology gene sets are listed in descending order of NES.

Supplementary Data 5

Differentially expressed genes between LS AML and LC AML. Genes are listed in ascending order of adjusted p-values regarding the difference in expression.

Supplementary Data 6

Gene mutations and their valiant allele frequencies in LS AML samples. Two-tailed *p*-values were calculated by Fisher's exact test and EBCall (Empirical Baysian mutation Calling).
