# Supplementary material for: Multi-omics analysis identifies an M-MDSC-like immunosuppressive phenotype in lineage-switched AML with KMT2A rearrangement
Source: Nat Commun. 2025 Aug 26;16:7955. doi: 10.1038/s41467-025-63271-y (PMC12381044; doi:10.1038/s41467-025-63271-y)
Supplement: Supplementary file 9 — Reporting Summary [file 41467_2025_63271_MOESM9_ESM.pdf]

nature portfolio

Corresponding author(s): Itaru Kato and Junko Takita

Last updated by author(s): Jul 15, 2025

Reporting Summary

Nature Portfolio wishes to improve the reproducibility of the work that we publish. This form provides structure for consistency and transparency in reporting. For further information on Nature Portfolio policies, see our Editorial Policies and the Editorial Policy Checklist.

Statistics

For all statistical analyses, confirm that the following items are present in the figure legend, table legend, main text, or Methods section.

n/a	Confirmed	
☐	☒	The exact sample size (n) for each experimental group/condition, given as a discrete number and unit of measurement
☐	☒	A statement on whether measurements were taken from distinct samples or whether the same sample was measured repeatedly
☐	☒	The statistical test(s) used AND whether they are one- or two-sided<br>*Only common tests should be described solely by name; describe more complex techniques in the Methods section.*
☐	☒	A description of all covariates tested
☐	☒	A description of any assumptions or corrections, such as tests of normality and adjustment for multiple comparisons
☐	☒	A full description of the statistical parameters including central tendency (e.g. means) or other basic estimates (e.g. regression coefficient) AND variation (e.g. standard deviation) or associated estimates of uncertainty (e.g. confidence intervals)
☐	☒	For null hypothesis testing, the test statistic (e.g. F, t, r) with confidence intervals, effect sizes, degrees of freedom and P value noted<br>*Give P values as exact values whenever suitable.*
☒	☐	For Bayesian analysis, information on the choice of priors and Markov chain Monte Carlo settings
☒	☐	For hierarchical and complex designs, identification of the appropriate level for tests and full reporting of outcomes
☒	☐	Estimates of effect sizes (e.g. Cohen's d, Pearson's r), indicating how they were calculated

Our web collection on statistics for biologists contains articles on many of the points above.

Software and code

Policy information about availability of computer code

Data collection	Flow cytometry: BD FACSuite (1.0.6.5230)<br>Mass cytometry: CyTOF Software (7.0.8493)
Data analysis	Data analysis was performed in R (3.6.3, 4.2.1, 4.3.1 or 4.3.2). Softwares/packages used include:<br>(For RNA-seq data analysis) Genomon (2.6.2); STAR (2.7.11a); DESeq2 (1.36.0); GSEA (4.3.2); ConsensusClusterPlus (1.50.0), ClustVis (https://biit.cs.ut.ee/clustvis/); pheatmap (1.0.12); ggVolcanoR (1.0.2)<br>(For single-cell RNA-seq analysis) Metascape (https://metascape.org/), SPRING (https://kleintools.hms.harvard.edu/tools/spring.html)<br>(For whole exome sequencing data analysis) Genomon (2.6.2); EBFilter (0.2.1; https://github.com/Genomon-Project/EBFilter)<br><br>Flow cytometry and mass cytometry data were analyzed using Cytobank (https://www.cytobank.org), FlowJo software (10.8.1) and GraphPad Prism 9.5.1).

For manuscripts utilizing custom algorithms or software that are central to the research but not yet described in published literature, software must be made available to editors and reviewers. We strongly encourage code deposition in a community repository (e.g. GitHub). See the Nature Portfolio guidelines for submitting code & software for further information.

Data

Policy information about availability of data

All manuscripts must include a data availability statement. This statement should provide the following information, where applicable:

- Accession codes, unique identifiers, or web links for publicly available datasets
- A description of any restrictions on data availability
- For clinical datasets or third party data, please ensure that the statement adheres to our policy

Whole exome sequencing data and RNA sequencing data for LS AML and LC AML (KMT2A-r AML), obtained from the in-house cohort, are available at the Japanese Genome-phenotype Archive (JGA) managed by the DNA Data Bank of Japan (DDBJ) (https://www.ddbj.nig.ac.jp/index-e.html) under accession number JGAS000631. The results published herein are in part based upon data generated by the Therapeutically Applicable Research to Generate Effective Treatments (TARGET) (https://www.cancer.gov/ccg/research/genome-sequencing/target) initiative, phs000218. The data used for analysis are available at the Genomic Data Commons (https://portal.gdc.cancer.gov).
The mass cytometry data have been deposited in Zenodo (ID:15909979 [10.5281/zenodo.15909979]).

Research involving human participants, their data, or biological material

Policy information about studies with human participants or human data. See also policy information about sex, gender (identity/presentation), and sexual orientation and race, ethnicity and racism.

Reporting on sex and gender	Sex and gender were not considered in the study design, and sex of patients was determined based on self-report. For the detailed data, please refer to supplementary table 2.
Reporting on race, ethnicity, or other socially relevant groupings	All patients included in our in-house cohort were Asian.
Population characteristics	For the information about population characteristics, please refer to supplementary table 2.
Recruitment	We have recruited cases of the relevant disease in our institution or affiliated hospitals in Japan.
Ethics oversight	This study was approved by the Kyoto University Hospital Ethical Board (G-1030, R-2831) and related institutions. The patients or their guardians provided informed consent for sample collection.

Note that full information on the approval of the study protocol must also be provided in the manuscript.

Field-specific reporting

Please select the one below that is the best fit for your research. If you are not sure, read the appropriate sections before making your selection.

☒ Life sciences ☐ Behavioural & social sciences ☐ Ecological, evolutionary & environmental sciences

For a reference copy of the document with all sections, see nature.com/documents/nr-reporting-summary-flat.pdf

Life sciences study design

All studies must disclose on these points even when the disclosure is negative.

Sample size	No prior sample size calculation was performed. Considering the rarity of the disease, cases were included on the basis of sample availability.
Data exclusions	There was no data exclusion in this study.
Replication	All in vitro experiments were repeated three times, with at least three biological replicates.
Randomization	Patient randomization was not relevant for this study.
Blinding	Investigators were not blind to experimental groups as knowledge of cell type identities was necessary to perform the experiments and analyses.

Reporting for specific materials, systems and methods

We require information from authors about some types of materials, experimental systems and methods used in many studies. Here, indicate whether each material, system or method listed is relevant to your study. If you are not sure if a list item applies to your research, read the appropriate section before selecting a response.

Materials & experimental systems

n/a	Involved in the study
☐	☒ Antibodies
☒	☐ Eukaryotic cell lines
☒	☐ Palaeontology and archaeology
☒	☐ Animals and other organisms
☒	☐ Clinical data
☒	☐ Dual use research of concern
☒	☐ Plants

Methods

n/a	Involved in the study
☒	☐ ChIP-seq
☐	☒ Flow cytometry
☒	☐ MRI-based neuroimaging

Antibodies

Antibodies used

Antibody used in this study are described in the Methods, supplementary table 6 and supplementary table 9. The details are also listed below.

(For mass cytometry analysis)

CD45(HI30)-Y89 (Standard BioTools, 3089003B, 2:100)
CD235ab(HIR2) (BioLegend, 306602, 1:100)
CD86(BU63) (BioLegend, 374202, 4:100)
CD196(G034E3)-141Pr (Standard BioTools, 3141003A, 1:100)
CD19(HIB19)-142Nd (Standard BioTools, 3142001B, 1:100)
HLA-DR(L243)-143Nd (Standard BioTools, 3143013B, 0.5:100)
CD38(HIT2)-144Nd (Standard BioTools, 3144014B, 1:100)
CD4(RPA-T4)-145Nd (Standard BioTools, 3145001B, 1:100)
CD8a(RPA-T8)-146Nd (Standard BioTools, 3146001B, 1:100)
CD20(2H7)-147Sm (Standard BioTools, 3147001B, 1:100)
CD274(29E.2A3)-148Nd (Standard BioTools, 3148017B, 1:100)
CD25(2A3)-149Sm (Standard BioTools, 3149010B, 1:100)
CD134(ACT35)-150Nd (Standard BioTools, 3150023B, 1:100)
CD14(M5E2)-151Eu (Standard BioTools, 3151009B, 1:100)
CD13(WM15)-152Sm (Standard BioTools, 3152003B, 1:100)
Tim-3(F38-2E2)-153Eu (Standard BioTools, 3153008B, 1:100)
CD3(UCHT1)-154Sm (Standard BioTools, 3154003B, 1:100)
CD27(L128)-155Gd (Standard BioTools, 3155001B, 0.5:100)
CD183(G025H7)-156Gd (Standard BioTools, 3156004B, 2:100)
CD10(HI10a)-158Gd (Standard BioTools, 3158011B, 0.5:100)
CD22(HIB22)-159Tb (Standard BioTools, 3159005B, 1:100)
CD28(CD28.2)-160Gd (Standard BioTools, 3160003B, 2:100)
CD152(14D3)-161Dy (Standard BioTools, 3161004B, 1:100)
Foxp3(236A/E7) (eBioscience, 14-4777-82, 2:100)
CD56(NCAM16.2)-163Dy (Standard BioTools, 3163007B, 0.5:100)
CD15(W6D3)-164Dy (Standard BioTools, 3164001B, 1:100)
CD223(11C3C65)-165Ho (Standard BioTools, 3165037B, 1:100)
CD34(581)-166Er (Standard BioTools, 3166012B, 1:100)
CD197(G043H7)167Er (Standard BioTools, 3167009A, 1:100)
CD357(621)-168Er (BioLegend, 311602, 2:100)
CD33(WM53)-169Tm (Standard BioTools, 3169010B, 0.5:100)
CD45RA(HI100)-170Er (Standard BioTools, 3170010B, 0.5:100)
CD185(RF8B2)-171Yb (Standard BioTools, 3171014B, 2:100)
CD273(24F.10C12)-172Yb (Standard BioTools, 3172014B, 1:100)
CD137(4B4-1)-173Yb (Standard BioTools, 3173015B, 1:100)
CD279(EH12.2H7)-174Yb (Standard BioTools, 3174020B, 2:100)
CD194(L291H4)-175Lu (Standard BioTools, 3175035A, 0.5:100)
CD127(A019D5)-176Yb (Standard BioTools, 3176004B, 0.5:100)
CD11b(ICRF44)-209Bi (Standard BioTools, 3209003B, 0.5:100)

(For flow cytometry analysis)

CD3(UCHT1)-BV421 (BioLegend, 300434, 1:200)
CD4(RPA-T4)-APC (BioLegend, 300514, 1:200)
CD4(RPA-T4)-FITC (BioLegend, 300506, 1:100)
CD25(2A3)-BV421 (BD, 564033, 2:100)
CD127(A019D5)-PerCP/Cy5.5 (BioLegend, 351322, 1:100)
CD45RA(HI100)-APC (BioLegend, 304112, 1:100)
CD45RA(HI100)-PerCP/Cy5.5 (BioLegend, 304122, 1:100)
Foxp3(236A/E7)-PE (eBioscience, 12-4777-42, 5:100)
IFN-γ(B27)-BV421 (BioLegend, 506538, 5:100)

CD45(HI30)-PerCP/Cy5.5 (BioLegend, 304028, 1:100)

CD15(W6D3)-BV421 (BioLegend, 323040, 1:100)
CD11b(M1/70)-FITC (BioLegend, 101206, 2:100)
CD14(M5E2)-APC (BioLegend, 301808, 4:100)
HLA-DR(L243)-PE (BioLegend, 307606, 1:100)

Validation

All antibodies are commercially available and validated by manufacturers. The validation of each primary antibody for the reactive species and applications are described below.

(For mass cytometry analysis)

CD45(HI30)-Y89 (Standard BioTools, 3089003B) - Reactive species: Human, Application: Mass cytometry
CD235ab(HIR2) (BioLegend, 306602) - Reactive species: Human, Application: Flow cytometry, CyTOF, Immunohistochemistry-Paraffin, Spatial Biology
CD86(BU63) (BioLegend, 374202) - Reactive species: Human, Application: Flow cytometry, Blocking, Immunohistochemistry, Western blotting
CD196(G034E3)-141Pr (Standard BioTools, 3141003A) - Reactive species: Human, Application: Mass cytometry
CD19(HIB19)-142Nd (Standard BioTools, 3142001B) - Reactive species: Human, Application: Mass cytometry
HLA-DR(L243)-143Nd (Standard BioTools, 3143013B) - Reactive species: Human, Application: Mass cytometry
CD38(HIT2)-144Nd (Standard BioTools, 3144014B) - Reactive species: Human, Application: Mass cytometry
CD4(RPA-T4)-145Nd (Standard BioTools, 3145001B) - Reactive species: Human, Application: Mass cytometry
CD8a(RPA-T8)-146Nd (Standard BioTools, 3146001B) - Reactive species: Human, Application: Mass cytometry
CD20(2H7)-147Sm (Standard BioTools, 3147001B) - Reactive species: Human, Application: Mass cytometry
CD274(29E.2A3)-148Nd (Standard BioTools, 3148017B) - Reactive species: Human, Application: Mass cytometry
CD25(2A3)-149Sm (Standard BioTools, 3149010B) - Reactive species: Human, Application: Mass cytometry
CD134(ACT35)-150Nd (Standard BioTools, 3150023B) - Reactive species: Human, Application: Mass cytometry
CD14(M5E2)-151Eu (Standard BioTools, 3151009B) - Reactive species: Human, Application: Mass cytometry
CD13(WM15)-152Sm (Standard BioTools, 3152003B) - Reactive species: Human, Application: Mass cytometry
Tim-3(F38-2E2)-153Eu (Standard BioTools, 3153008B) - Reactive species: Human, Application: Mass cytometry
CD3(UCHT1)-154Sm (Standard BioTools, 3154003B) - Reactive species: Human, Application: Mass cytometry
CD27(L128)-155Gd (Standard BioTools, 3155001B) - Reactive species: Human, Application: Mass cytometry
CD183(G025H7)-156Gd (Standard BioTools, 3156004B) - Reactive species: Human, Application: Mass cytometry
CD10(HI10a)-158Gd (Standard BioTools, 3158011B) - Reactive species: Human, Application: Mass cytometry
CD22(HIB22)-159Tb (Standard BioTools, 3159005B) - Reactive species: Human, Application: Mass cytometry
CD28(CD28.2)-160Gd (Standard BioTools, 3160003B) - Reactive species: Human, Application: Mass cytometry
CD152(14D3)-161Dy (Standard BioTools, 3161004B) - Reactive species: Human, Application: Mass cytometry
Foxp3(236A/E7) (eBioscience, 14-4777-82) - Reactive species: Human, Non-human primate, Rhesus monkey, Application: Western blotting, Immunohistochemistry (Paraffin, Frozen, Immunocytochemistry), Flow cytometry, Immunoprecipitation, Radioimmune assays
CD56(NCAM16.2)-163Dy (Standard BioTools, 3163007B) - Reactive species: Human, Application: Mass cytometry
CD15(W6D3)-164Dy (Standard BioTools, 3164001B) - Reactive species: Human, Application: Mass cytometry
CD223(11C3C65)-165Ho (Standard BioTools, 3165037B) - Reactive species: Human, Application: Mass cytometry
CD34(581)-166Er (Standard BioTools, 3166012B) - Reactive species: Human, Application: Mass cytometry
CD197(G043H7)167Er (Standard BioTools, 3167009A) - Reactive species: Human, Application: Mass cytometry
CD357(621)-168Er (BioLegend, 311602) - Reactive species: Human, African Green, Baboon, Cynomolgus, Rhesus, Application: Flow cytometry
CD33(WM53)-169Tm (Standard BioTools, 3169010B) - Reactive species: Human, Application: Mass cytometry
CD45RA(HI100)-170Er (Standard BioTools, 3170010B) - Reactive species: Human, Application: Mass cytometry
CD185(RF8B2)-171Yb (Standard BioTools, 3171014B) - Reactive species: Human, Application: Mass cytometry
CD273(24F.10C12)-172Yb (Standard BioTools, 3172014B) - Reactive species: Human, Application: Mass cytometry
CD137(4B4-1)-173Yb (Standard BioTools, 3173015B) - Reactive species: Human, Application: Mass cytometry
CD279(EH12.2H7)-174Yb (Standard BioTools, 3174020B) - Reactive species: Human, Application: Mass cytometry
CD194(L291H4)-175Lu (Standard BioTools, 3175035A) - Reactive species: Human, Application: Mass cytometry
CD127(A019D5)-176Yb (Standard BioTools, 3176004B) - Reactive species: Human, Application: Mass cytometry
CD11b(ICRF44)-209Bi (Standard BioTools, 3209003B) - Reactive species: Human, Application: Mass cytometry

(For flow cytometry analysis)

CD3(UCHT1)-BV421 (BioLegend, 300434) - Reactive species: Human, Chimpanzee, Application: Flow cytometry, Immunohistochemistry (Frozen), Immunocytochemistry
CD4(RPA-T4)-APC (BioLegend, 300514) - Reactive species: Human, Chimpanzee, Application: Flow cytometry
CD4(RPA-T4)-FITC (BioLegend, 300506) - Reactive species: Human, Chimpanzee, Application: Flow cytometry, Spatial biology
CD25(2A3)-BV421 (BD, 564033) - Reactive species: Human, Application: Flow cytometry
CD127(A019D5)-PerCP/Cy5.5 (BioLegend, 351322) - Reactive species: Human, African Green, Baboon, Cynomolgus, Rhesus, Application: Flow cytometry
CD45RA(HI100)-APC (BioLegend, 304112)- Reactive species: Human, Chimpanzee, Application: Flow cytometry
CD45RA(HI100)-PerCP/Cy5.5 (BioLegend, 304122)- Reactive species: Human, Chimpanzee, Application: Flow cytometry, Spatial biology
Foxp3(236A/E7)-PE (eBioscience, 12-4777-42)- Reactive species: Human, Non-human primate, Rhesus monkey, Baboon, Mouse, Application: Immunohistochemistry (Paraffin, Immunocytochemistry), Flow cytometry, Luminex
IFN-γ(B27)-BV421 (BioLegend, 506538)- Reactive species: Human, Application: Intracellular staining for flow cytometry

CD45(HI30)-PerCP/Cy5.5 (BioLegend, 304028) - Reactive species: Human, Chimpanzee, Application: Flow cytometry, Spatial biology
CD15(W6D3)-BV421 (BioLegend, 323040) - Reactive species: Human, Application: Flow cytometry
CD11b(M1/70)-FITC (BioLegend, 101206) - Reactive species: Mouse, Human, Cynomolgus, Rhesus, Application: Flow cytometry
CD14(M5E2)-APC (BioLegend, 301808) - Reactive species: Human, Cynomolgus, Rhesus, Application: Flow cytometry

HLA-DR(L243)-PE (BioLegend, 307606) - Reactive species: Human, Cynomolgus, Rhesus, Application: Flow cytometry

Plants

Seed stocks	Not applicable.
Novel plant genotypes	Not applicable.
Authentication	Not applicable.

Flow Cytometry

Plots

Confirm that:

☒ The axis labels state the marker and fluorochrome used (e.g. CD4-FITC).

☒ The axis scales are clearly visible. Include numbers along axes only for bottom left plot of group (a 'group' is an analysis of identical markers).

☒ All plots are contour plots with outliers or pseudocolor plots.

☒ A numerical value for number of cells or percentage (with statistics) is provided.

Methodology

Sample preparation	For cell surface marker staining, cells were stained with fluoro-conjugated antibodies at room temperature for 30 min. Fixation and permeabilization were performed using FOXP3/Transcription Factor Staining Buffer Set (eBioscience). Intracelllar staining was conducted using anti-human Foxp3 or IFN-γ antibodies at 4 °c for 30min. After staining, the samples were washed twice in 1×perm buffer (eBioscience), and were suspended with PBS containing 2% PBS.
Instrument	Cells were analyzed on a FACSLyric (BD Biosciences).
Software	Data were collected using FACSuite software and were analyzed with FlowJo or Cytobank.
Cell population abundance	After cell sorting using FACSAria II, the purity of the target cell fractions were confirmed to be 90% or higher.
Gating strategy	Lymphocytes were gated by size exclusion (FSC-A/SSC-A) followed by doublet exclusion (FSC-H/FSC-W). Live cells were distinguished as non-stained cells by Fixable Viability Dye eFluor780. Subsequently, in the T cell suppression assay, responder T cells were gated as CD4+, and their devision was calculated by CFSE fluorescence intensity, as well as measuring intracellular IFN-γ intensity. In the Treg co-culture assay, CD4+ T cells were gated, followed by effector Tregs detection as a CD25+CD127-CD45RA-Foxp3high population.

☒ Tick this box to confirm that a figure exemplifying the gating strategy is provided in the Supplementary Information.
